# Does HPV Subtype Predict Outcomes in Head and Neck Cancers?

**DOI:** 10.1155/2021/6672373

**Published:** 2021-02-09

**Authors:** Hedyeh Ziai, Andrew Warner, Neil Mundi, Krupal Patel, Eun Jae Chung, Christopher J. Howlett, Paul Plantinga, John Yoo, S. Danielle MacNeil, Kevin Fung, Joe S. Mymryk, John W. Barrett, David A. Palma, Anthony C. Nichols

**Affiliations:** ^1^Department of Otolaryngology-Head and Neck Surgery, Western University, ON, Canada; ^2^Department of Oncology, Western University, ON, Canada; ^3^Department of Otolaryngology, Seoul University, Seoul, Republic of Korea; ^4^Department of Pathology, Western University, ON, Canada; ^5^Department of Microbiology & Immunology, Western University, ON, Canada

## Abstract

**Background:**

Recently, reanalysis of The Cancer Genome Atlas study demonstrated that human papillomavirus (HPV) genotypes in head and neck cancers other than HPV-16 have inferior survival to HPV-16-positive tumors. We aimed to examine the association of HPV subtypes and survival in a large cohort of patient samples from our institution.

**Methods:**

Fresh frozen primary site biopsy samples were collected either in clinic or at the time of surgery. Patient demographic, staging, and survival data were also collected. Tumors were tested for HPV subtypes by quantitative polymerase chain reaction (qPCR). Univariable and multivariable analyses were performed using Cox proportional hazards regression.

**Results:**

280 patient biopsy samples were collected between 2011 and 2017. Mean ± standard deviation (SD) age was 61.9 ± 11.1 years and most patients (78%) were male. The majority of cancers were of the oral cavity (60%) or oropharynx (25%) and 30% had HPV-positive disease. Median follow-up was 3.76 years and 96/280 patients (34%) developed recurrences. Patients with p16-positive versus negative disease had significantly improved 5-year overall survival (OS, 77.6% vs. 53.3%; *p*=0.009) and progression-free survival (PFS, 67.3% vs. 41.0%, *p*=0.006). Similarly improved 5-year OS and PFS were observed for patients with HPV-positive versus negative disease (65.0% vs. 55.0%, *p*=0.084; 53.3% vs. 43.2%, *p*=0.072, resp.). Patients with HPV-16 compared to other HPV diseases had worse 5-year OS and PFS (62.1% vs. 88.9%, *p*=0.273; 49.0% vs. 88.9%, *p*=0.081, resp.).

**Conclusions:**

In contrast to the data derived from The Cancer Genome Atlas, patients with HPV-16 tumors trended towards decreased PFS and OS compared with tumors driven by other HPV genotypes. Further larger multi-institutional studies are necessary to understand the relationship between other HPV genotypes and survival in head and neck squamous cell carcinomas.

## 1. Introduction

There has been a dramatic rise in a subset of head and neck squamous cell carcinomas (HNSCC) due to increasing rates of oral infection with human papillomavirus (HPV) leading to HPV-associated head and neck cancers [1, 2]. Although the majority of these HPV-associated tumors are located in the oropharynx, a small proportion of tumors arising from other sites including the oral cavity, hypopharynx, larynx, and nasopharynx have also been shown to be HPV-related. Although there are nearly 200 HPV genotypes, only several high-risk HPV genotypes are considered causative agents in head and neck cancers [3–5]. Of these, HPV-16 is the most commonly detected; a recent meta-analysis suggested that 82% of HPV-positive head and neck cancers were attributable to the HPV-16 genotype [6].

HPV and its surrogate marker p16 have been definitively shown to be independent prognostic biomarkers for improved survival for cancers arising within the oropharynx in the context of prospective randomized controlled trials [7–10]. Five-year overall survival rates exceed 80% for patients with locoregionally advanced HPV-associated/*p*16-positive oropharyngeal squamous cell carcinomas (OPSCC) treated with radiation and chemotherapy compared with approximately 40% for those with HPV-negative tumors treated with similar regimens [9, 11]. Since HPV-positive patients fare so well, there is great interest in deescalating treatment for this patient population, with the goal of decreasing treatment toxicity while maintaining cure rates [9, 12–27].

However, significant questions surrounding the role of HPV in HNSCC remain, including particularly the following: (1) whether HPV plays a prognostic role in subsites other than the oropharynx, and (2) if HPV subtypes other than type 16 have the same prognostic significance. However, there have been recent high-quality data addressing each of these questions that were previously limited by smaller datasets. In contrast with many prior studies showing no difference in outcome outside of the oropharynx, Li and colleagues utilized the US national cancer database (NCDB) data and analyzed all HNSCC patients (*n* = 41,950) with established HPV status (PMID 29801040). They found that patients with HPV-positive oral, oropharyngeal, laryngeal, and hypopharyngeal tumors faired better than HPV-negative patients (oral cavity (hazard ratio [HR] = 0.76; 95% CI, 0.66–0.87), oropharynx (HR = 0.44; 95% CI, 0.41–0.47), hypopharynx (HR = 0.59; 95% CI, 0.45–0.77), and larynx (HR = 0.71; 95% CI, 0.59–0.85)). Unfortunately, the HPV type for each patient was not available to draw type specific conclusions.

To address the HPV type question, Bratman and colleagues reanalyzed The Cancer Genome Atlas (TCGA) cohort and utilized viral reads derived from the RNA sequencing to determine definitive HPV status and HPV type. [3] We note that TCGA cohort reflects a surgical series as a resection specimen was required for inclusion [1]. The subsite breakdown is as follows: 311 oral cavity, 79 oropharyngeal, 115 laryngeal, and 10 hypopharyngeal cancers. Of 515 tumors, HPV oncoprotein transcripts were observed in 73 tumors (14%). Sixty-one of the 73 HPV-positive tumors (84%) expressed HPV-16 oncogenes, while the remainder were attributed to types 33 (*n* = 8), 35 (*n* = 3), and 56 (*n* = 1). This analysis revealed that HPV-tumor types other than HPV-16 had inferior survival when compared to their counterparts with HPV-16-positive disease, with similar survival rates to those seen in HPV-negative HNSCC. This suggests that patients with these genotypes may be inappropriate candidates for treatment deintensification [28, 29] and should proceed with traditional non-HPV-associated HNSCC treatment algorithms.

The study by Bratman had strengths such as relatively large sample size and definitive HPV detection and subtyping through RNA sequencing; however, it also had weakness including lack of an independent validation cohort, imperfect TCGA survival data [2], and the fact that 30% of patients did not receive treatment that complied with the NCCN guidelines [3]. In an attempt to externally validate the findings of the Bratman study, we analyzed a prospectively collected large cohort of patients with head and neck squamous cell carcinomas to determine whether non-HPV-16 genotypes may predict for more aggressive disease and to determine if the association between HPV type and survival was reproducible.

## 2. Materials and Methods

### 2.1. Patient Population

Patients with head and neck squamous cell cancer set to undergo treatment with curative intent (with either primary radiation or primary surgery) were prospectively enrolled through the head and neck cancer clinic at London Health Sciences Centre. Patients with distant metastatic disease, treated with palliative intent, with known recurrence prior to biopsy sampling, or with missing HPV testing data were excluded. Clinical data was collected, including age at diagnosis, use of tobacco and alcohol, AJCC TNM staging (7^th^ edition), treatment regimen, and posttreatment follow-up information. P16 testing was performed on formalin fixed samples for the majority of patients (59/70) with oropharyngeal cancer primaries and a subset of other cases (27/210) as part of routine clinical care.

### 2.2. Tumor Collection

Fresh tumor was harvested either from a biopsy in clinic or from the center of the ablation specimen after the resection was complete, with care taken not to disturb the margins. Tumor cellularity of >70% was confirmed by frozen section analysis. The tumor was placed on ice and transported to the research laboratory, where a portion was frozen, and another piece underwent immediate DNA extraction using Qiagen kits (Cat#: 69504).

### 2.3. qPCR Analysis of HPV Status

In an effort to screen clinical samples for the presence of human papillomavirus (HPV), we designed a multiplex quantitative PCR to identify those samples that were HPV-positive and to confirm the HPV type in the positive samples. Tumor genomic DNA (gDNA) samples were screened with primer sets designed using MacVector Ver.15 software (Primer3) to amplify small stretches within the E6/*E*7 regions of HPV −16, −18, −33, −35, and −56. We also included HPV type 18 in our analysis as we have previously identified its presence in head and neck cancer samples [29] despite the fact that it was not seen in the TCGA dataset. GAPDH was used as the housekeeping gene/internal control. The fluorescent dyes (Cy5: GAPDH; Joe: HPV−16, and −33; ROX: HPV −18 and −35; FAM: HPV-56) were conjugated to a probe, designed to bind within the amplicon for each target gene. The HPV−16 and −18 and GAPDH primer/probe sets have been described and optimized previously. [29, 30] We designed primer/probe sets (Supplemental Table 1) against a 115 nucleotide (nt) fragment within exon 6 of GAPDH (internal control), a 110 nt region across E6-E7 of HPV-16, a 137 nt fragment across the HPV-18 E6-E7 region, a 68 nt fragment across E6-E7 of HPV-56, an 80 nt fragment across E6-E7 of HPV-33, and a 99 nt fragment across E6-E7 of HPV-35. Each sample was run first with the GAPDH, HPV-16, HPV-18, and HPV-56 primer/probe mix and then the same samples were run again with GAPDH, HPV-33, HPV-35, and HPV-56 mix.

qPCR reactions (10 *μ*l) were prepared using the QuantiTect multiplex PCR no ROX kit (QIAGEN) with 0.2 *μ*l of template gDNA. A Stratagene Mx3000P was used for performing qPCR with the following conditions: one cycle of heat inactivation/enzyme activation at 95°C for 15 min, 40 cycles of denaturation at 94°C for 60 seconds, and annealing/extension at 60°C for 90 seconds.

Genomic DNA from CaSki and HeLa cell lines were used as HPV-16 and −18 positive controls, respectively. HPV-33, −35, and −56 plasmids were used as positive controls for confirmation of the presence of HPV-33, −35, and −56 DNA, respectively. No template control (NTC) was used to determine the baseline for each probe to determine threshold cycle (Ct) values. Samples with GAPDH Ct ≥ 35 indicated poor gDNA yield. Signals for any fluorescent dye greater than Ct ≥ 35 were considered questionable and were repeated before an evaluation was made.

### 2.4. Statistical Analysis

Descriptive statistics were generated for all patients and stratified by HPV status (HPV-16, HPV-other vs. HPV-negative), compared using the Chi-square test or Fisher's Exact test, analysis of variance (ANOVA), or Kruskal–Wallis test as appropriate. Overall survival was calculated as the time from date of consult to date of last follow-up and/or death (any cause), whichever comes first. Progression-free survival was calculated as the time from date of consult to date of last follow-up and/or recurrence and/or death (any cause), whichever comes first. The Kaplan–Meier estimates were generated for OS and PFS for all patients, patients receiving primary surgical treatment (excluding nonsurgical primary treatment such as organ preservation chemoradiotherapy) and patients with oropharyngeal cancer only, stratified by HPV and p16 status and compared using the log-rank test. Univariable and multivariable Cox proportional hazards regression was performed on all patients to identify significant predictors of OS and PFS. All eligible variables with univariable *p* values <0.05 were incorporated into a multivariable regression model and sequentially removed using backward elimination techniques until all remaining covariates had *p* values <0.05. All statistical analysis was performed using SAS version 9.4 software (SAS Institute, Cary NC) using two-sided statistical testing at the 0.05 significance level.

## 3. Results

### 3.1. Baseline Characteristics

Between 2011 and 2017, biopsy samples from 280 patients with HNSCC meeting our inclusion criteria were obtained. Baseline patient and tumor characteristics are shown in Table 1. Mean ± SD age was 61.9 ± 11.1 years and the majority of patients were male (77.5%) with cancers of the oral cavity (60.4%) or oropharynx (25.0%). Most patients were treated with primary surgery (76.1%). HPV-positive disease was identified in 84 patients (30.0%), with HPV-16 in 75 (26.8%), HPV-18 in 5 (1.8%), HPV-33 in 1 (0.4%), and HPV-35 in 3 (1.1%), confirmed by HPV testing of genomic DNA. Other HPV diseases (defined as HPV-positive disease for HPV-16-negative patients) were identified in 9 patients (3.2%). P16 status was determined for 86 patients in the context of clinical care. Fifty of 86 of these (58%) were positive of which 41/46 (89.1%) were HPV-16-positive, 5/6 (83.3%) were positive for other HPV types, and 4/34 (11.7%) were HPV-negative.

Compared to their HPV-negative counterparts, HPV-16 and other HPV-positive patients tended to be younger (*p*=0.034) and less likely to have T4 disease (*p*=0.113), perineural invasion (*p*=0.071), and extranodal extension (*p*=0.171). HPV-16-positive patients received more nonsurgical treatment (*p* < 0.001) and less adjuvant treatment (*p* < 0.001) compared to HPV-negative and other HPV patients. In addition, HPV-16-positive and other HPV patients were more likely to have oropharyngeal primaries compared to HPV-negative patients (64.0%, 44.4%, and 9.2% resp.) and less likely to have oral cavity primaries (20.0%, 44.4%, and 76.5%, resp., *p* < 0.001).

### 3.2. Survival Outcomes

**Figure 1:**
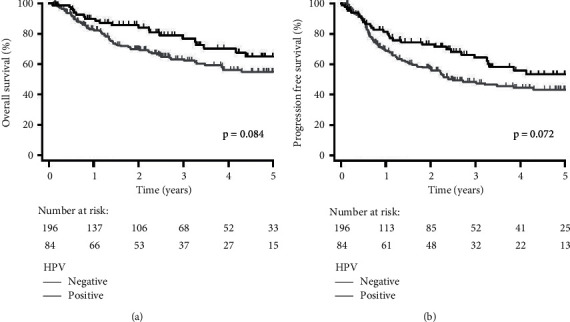
Kaplan–Meier plots stratified by HPV status (positive vs. negative) for (a) overall survival and (b) progression-free survival for all patients (*n* = 280).

**Figure 2:**
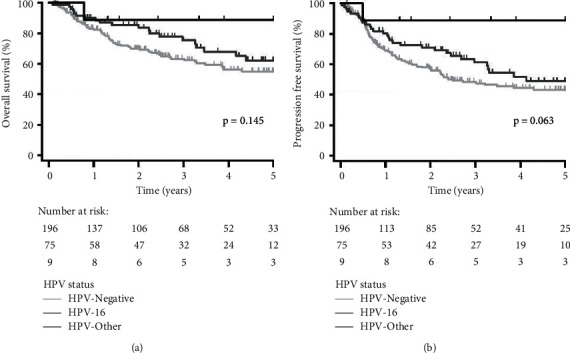
Kaplan–Meier plots stratified by HPV status (HPV-16 vs. HPV-other vs. HPV-negative) for (a) overall survival and (b) progression-free survival for all patients (*n* = 280).

**Figure 3:**
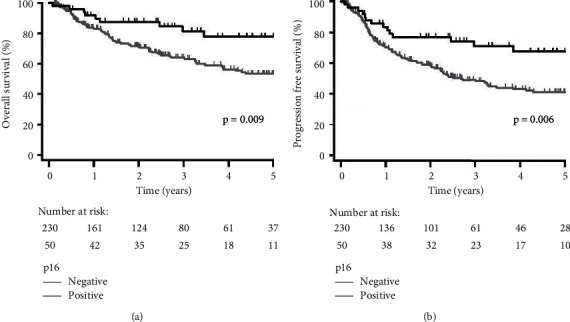
Kaplan–Meier plots stratified by p16 status (positive vs. negative/unknown) for (a) overall survival and (b) progression-free survival for all patients (*n* = 280).

Median follow-up was 3.76 years (95% confidence interval [CI]: 2.87–4.21). Ninety-two patients (32.9%) were deceased and 96 patients (34.3%) had developed recurrences. Five-year OS and PFS for all patients were 57.9% and 46.3%, respectively. Compared to HPV-negative patients, HPV-positive patients had improved 5-year OS and PFS (65.0% vs. 55.0%, *p*=0.084; 53.3% vs. 43.2%, *p*=0.072, resp.); however, this was not significant (Figure 1). Patients with HPV-16 compared to other HPV had worse 5-year OS and PFS (62.1% vs. 88.9%, *p*=0.273; 49.0% vs. 88.9%, *p*=0.081, resp.). This remained not significant for both OS and PFS after incorporating HPV-negative patients (OS: *p*=0.145; PFS: *p*=0.063, Figure 2). Fewer recurrences developed for patients with other HPV diseases (11.1%) compared to HPV-negative (35.2%) and HPV-16-positive disease (34.7%); however, this was not significant (*p*=0.329). In contrast, patients with p16-positive disease had significantly better 5-year OS and PFS compared to patients with p16-negative/*p*16 unknown disease (77.6% vs. 53.3%, *p*=0.009; 67.3% vs. 41.0%, *p*=0.006, resp., Figure 3).

### 3.3. Cox Proportional Hazards Regression

Results from univariable and multivariable Cox proportional hazards regression models are shown in Table 2. The univariable analysis identified older age (hazard ratio [HR] per 5 years: 1.12, *p*=0.018), alcohol abuse (HR: 1.67, *p*=0.015), T4 versus T1-T2 disease (HR: 2.64, *p* < 0.001), N2-N3 versus N0 disease (HR: 2.41, *p* < 0.001), stage IV versus stages I-II disease (HR: 3.11, *p*=0.001), perineural invasion (HR: 2.58, *p* < 0.001), extranodal extension (HR: 2.92, *p* < 0.001), and lymphovascular invasion (HR: 2.81, *p* < 0.001) as significant predictors of worse OS. p16-positive disease was significantly predictive of better OS (HR: 0.41, *p*=0.011). Stage IV versus stages I-II disease (HR: 4.67, *p* < 0.001) and perineural invasion (HR: 2.53, *p* < 0.001) remained significantly predictive of worse OS from the multivariable analysis.

Similarly for PFS, univariable analysis identified T4 versus T1-T2 disease (HR: 1.70, *p*=0.011), N2-N3 versus N0 disease (HR: 1.56, *p*=0.020), perineural invasion (HR: 1.91, *p*=0.002), extranodal extension (HR: 2.05, *p*=0.003), lymphovascular invasion (HR: 1.72, *p*=0.016), and receiving neoadjuvant treatment (HR: 2.84, *p*=0.001) as significant predictors of inferior PFS. p16-positive disease was significantly predictive of improved PFS (HR: 0.48, *p*=0.008). From the multivariable analysis, T4 versus T1-T2 disease (HR: 2.01, *p*=0.006), N2-N3 versus N0 disease (HR: 2.00, *p*=0.003), perineural invasion (HR: 1.60, *p*=0.034) and receiving neoadjuvant treatment (HR: 4.28, *p* < 0.001) remained significantly predictive of worse PFS.

### 3.4. Primary Surgical Treatment

Two hundred and three patients received primary surgical treatment (excluding nonsurgical primary treatment such as organ preservation chemoradiotherapy). Compared to the initial cohort of 280 patients, these patients were more likely to have oral cavity cancer (79.8% vs. 60.4%), to have received adjuvant treatment (71.4% vs. 52.5%), and less likely to be HPV-positive (19.7% vs. 30.0%), HPV-16-positive (16.3% vs. 26.8%), and p16-positive (6.4% vs. 17.9%). Similarly compared to HPV-negative patients, HPV-positive patients had improved 5-year OS but similar 5-year PFS (62.1% vs. 54.1%, *p*=0.087; 44.4% vs. 46.8%, *p*=0.307, resp.), both not significant. HPV-16-positive patients compared to other HPV disease had worse 5-year OS and significantly worse 5-year PFS (52.1% vs. 100%, *p*=0.087; 29.4% vs. 100%, *p*=0.015, resp.); however, this was not significant after incorporating HPV-negative patients (OS: *p*=0.102; PFS: *p*=0.088). Although patients with p16-positive disease had improved 5-year OS and PFS compared to patients with p16-negative/unknown disease, this was no longer significant (69.9% vs. 54.0%; *p*=0.338; 63.5% vs. 44.5%, *p*=0.263, resp.).

### 3.5. Oropharyngeal Squamous Cell Carcinoma

**Figure 4:**
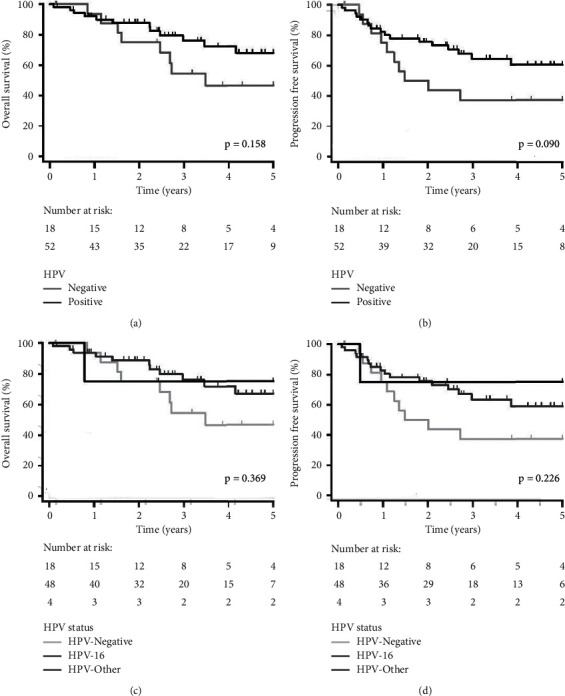
Kaplan–Meier plots stratified by HPV status (positive vs. negative) for (a) overall survival and (b) progression-free survival and stratified by HPV status (HPV-16 vs. HPV-other vs. HPV-negative) for (c) overall survival and (d) progression-free survival for patients with oropharyngeal cancer (*n* = 70).

Some studies have suggested that the improved survival associated with positive HPV status is not present for carcinomas in subsites other than the oropharynx [31]. For this reason, we repeated our analysis focusing on only cancers of the oropharynx. Patients with OPSCC represented 70 patients from the initial cohort. These patients were less likely to have T4 disease (14.5% vs. 37.8%) perineural invasion (27.3% vs. 39.3%) and to have received adjuvant treatment (12.9% vs. 52.5%), but more likely to have lymphovascular invasion (35.0% vs. 26.0%) and positive margins (16.7% vs. 6.7%) and to have received nonsurgical treatment (72.9% vs. 23.9%), have HPV-positive (74.3% vs. 30.0%), have HPV-16-positive (68.6% vs. 26.8%), and have p16-positive disease (64.3% vs. 17.9%). Improved 5-year OS and PFS were observed comparing HPV-positive to HPV-negative patients, although this was not significant (68.0% vs. 46.8%, *p*=0.158; 60.5% vs. 37.5%, *p*=0.090, resp., Figure 4). No significant differences were observed comparing HPV-16-positive patients to other HPV diseases (OS: *p*=0.976; PFS: *p*=0.727, resp.). Patients with p16-positive disease compared to p16-negative disease had significantly improved 5-year OS and PFS (79.9% vs. 30.5%, *p* < 0.001; 68.6% vs. 28.1%, *p*=0.003, resp.).

## 4. Discussion

The literature contains limited data regarding the prognostic implications of viral genotypes on oncologic control and survival. There are nearly 200 currently recognized genotypes of HPV, with several high-risk genotypes considered causative agents in OPSCC. These include HPV −16, −18, −33, −45, −52, and −58 [32]. We investigated the prognostic importance of distinct HPV genotypes within head and neck cancers. Our results support the findings of the low prevalence of HPV-18 in HNSCCs [6]. Our study did not corroborate previous findings suggesting that cases with HPV-16 genotype have superior survival versus other HPV genotypes in head and neck [3, 33–35] and other cancers [35, 36].

In contrast to the study by Bratman and colleagues, patients with HPV-16 disease did not experience superior survival to patients with disease due to other HPV genotypes. Rather, patients with disease due to the other type (HPV-18, −33, −35) appeared to fair better with a trend towards improved progression-free survival (Figure 2(b)). Patients with the p16-positive disease had significantly improved survival compared to those with p16-negative disease; however, this was not found to be significant in multivariable analysis. We acknowledge that this work is confounded by multiple subsites and heterogeneous treatments; however, we selected this population from our tumor bank to mirror the largely surgical series contained in the TCGA head and neck cohort. As the HPV-positive samples within the TCGA cohort are largely from the oropharynx (53 of 73), we repeated our analysis using only the samples from our cohort from this subsite. This also did not identify inferior outcomes for HPV-other disease. Thus, despite the parallels in patient population, we identified conflicting findings.

Our data support previous literature that p16 expression is strongly associated with improved survival, including sites other than the oropharynx (Figure 3). The prognostic significance of p16 expression in oropharyngeal SCCs has been well established [9, 37–39] and recently been shown in nonoropharyngeal HNSCC in the context of prospective trials [36]. Similar to the study by Chung et al., while p16 is strongly prognostic, HPV status was marginally correlated with survival in this heterogeneous population with only a trend towards improved progression-free, but not overall survival (Figure 1). The cause for this discrepancy is not clear. It is conceivable that some of these p16-positive cases are due to HPV types that we did not test for and this potentially represents a shortcoming of the study. Indeed, we limited our testing to the HPV subtypes identified in the Bratman study and HPV-18. However, the literature suggests that that is likely not the case [37]. There are likely yet to be understood molecular differences in these p16-positive, but HPV-negative, tumors that make them treatment sensitive. Thus, p16 likely represents a superior biomarker outside of the oropharynx.

Recent studies have suggested treatment deintensification for HPV-associated head and neck cancers as a promising therapeutic strategy with similar efficacy with decreased toxicity [28, 40–44]. The ultimate goal of treatment deintensification is to reduce the morbidity and functional implications, including dysphagia, speech-related toxicity, renal and hematologic toxicity [26, 45] while maintaining excellent oncologic outcomes. Patients with HPV-associated head and neck cancers are considered significantly more responsive to treatment than traditional tobacco- and alcohol-associated head and neck cancers [11, 26]. Maximizing quality of life by not overtreating patients is a uniformly supported goal with radiotherapy dose reduction and/or alterations in concurrent chemotherapy. Treatment deintensification is a reasonable goal in a select cohort of HPV-positive disease. Pretreatment prognostication is essential in treatment planning. In particular, the presence of HPV-associated disease and defining appropriate candidates for the deintensified treatment is critical in maintaining the excellent outcomes historically seen in patients with HPV-associated OPSCC. A positive immunohistochemical finding for p16 is often used as a surrogate for molecularly based HPV detection, based on the high concordance between these two biomarkers. However, p16 IHC analysis cannot distinguish between HPV genotypes. Our study failed to validate prior work done by Bratman et al. regarding improved survival outcomes for patients with HPV-16 disease. In contrast, we observed a nonsignificant inferior survival for HPV-16 genotype, compared to those with HPV-18, −33, and −35 disease; however, this requires future validation on a larger cohort given the relatively few numbers of patients with HPV-18/33/35 in our study (*n* = 9).

We suggest larger multi-institutional studies to determine the impact of HPV genotypes on patients with head and neck squamous cell carcinomas. Although HPV genotyping is not widely implemented in head and neck cancers, we believe that HPV genotyping should be routine in the management of HPV-positive head and neck cancers in the future. Our ability to risk stratify can be refined with the use of other potential adjunctive biomarkers to better assist physicians in selecting appropriate patients for deintensification. Future deintensification protocols should consider the pattern of relapse for the type of HPV-positive cancer. Patients with genotypes who have inferior survival rates comparable to those of HPV-negative head and neck cancers would then be excluded from consideration of treatment deintensification.

Although our patient data collection and tumor collection were prospective, there were limitations to our study. Our cohort involved a variety of tumor sites treated with heterogeneous regimens that could potentially obscure important findings. In addition, the number of tumors with other HPV genotypes was relatively small, which may limit the ability to detect significant differences. Given the rarity of other HPV types, large multi-institutional institutional efforts are likely needed to have sufficient power to conclusively answer this question.

## 5. Conclusions

To the best of our knowledge, this study is the first attempt to validate the findings by Bratman and colleagues on the prognostic significance of HPV genotypes in head and neck cancers. In contrast to their findings, patients with HPV-types other than 16 trended towards improved progression-free survival compared with HPV-16 related disease. Large, multi-institutional efforts are needed to conclusively determine the correlation of HPV type and survival in HNSCC.

## Figures and Tables

**Table 1:** Baseline patient and tumor characteristics stratified by HPV status (*n* = 280).

Characteristic	All patients (*n* = 280)	HPV status			*p* value
		HPV-negative (*n* = 196)	HPV-16 (*n* = 75)	HPV-other (*n* = 9)	
Age (years)–mean ± SD	61.9 ± 11.1	62.7 ± 11.1	60.8 ± 10.3	53.8 ± 13.2	**0.034**
Male–*n* (%)	217 (77.5)	148 (75.5)	61 (81.3)	8 (88.9)	0.418
T stage–*n* (%)					
T1	20 (7.2)	12 (6.2)	8 (10.8)	0 (0)	0.113
T2	96 (34.5)	61 (31.3)	31 (41.9)	4 (44.4)	
T3	57 (20.5)	38 (19.5)	17 (23.0)	2 (22.2)	
T4	105 (37.8)	84 (43.1)	18 (24.3)	3 (33.3)	
N stage–*n* (%)					
N0	124 (44.4)	91 (46.4)	27 (36.5)	6 (66.7)	0.221
N1	42 (15.1)	24 (12.2)	17 (23.0)	1 (11.1)	
N2	108 (38.7)	78 (39.8)	28 (37.8)	2 (22.2)	
N3	5 (1.8)	3 (1.5)	2 (2.7)	0 (0)	
Overall stage (AJCC 7^th^ edition)–*n* (%)					
I	8 (2.9)	5 (2.6)	3 (4.1)	0 (0)	0.415
II	47 (16.9)	34 (17.4)	10 (13.5)	3 (33.3)	
III	52 (18.6)	32 (16.3)	18 (24.3)	2 (22.2)	
IV	172 (61.7)	125 (63.8)	43 (58.1)	4 (44.4)	
Alcohol abuse–*n* (%)	110 (39.3)	83 (42.4)	24 (32.0)	3 (33.3)	0.276
Smoking pack-years–mean ± SD	27.2 ± 26.8	27.4 ± 23.2	26.2 ± 34.9	32.2 ± 26.7	0.345
Perineural invasion–*n* (%)	84 (39.3)	74 (43.0)	8 (23.5)	2 (25.0)	0.071
Extranodal extension–*n* (%)	43 (20.3)	39 (22.8)	3 (9.1)	1 (12.5)	0.171
Lymphovascular invasion–*n* (%)	54 (26.0)	44 (26.0)	9 (29.0)	1 (12.5)	0.636
Nonsurgical treatment–*n* (%)	67 (23.9)	23 (11.7)	42 (56.0)	2 (22.2)	**< 0.001**
Adjuvant treatment–*n* (%)	147 (52.5)	120 (61.2)	21 (28.0)	6 (66.7)	**< 0.001**
p16+–positive/number tested (%)	50/86 (58.1)	4/34 (11.7)	41/46 (89.1)	5/6 (83.3)	**< 0.001**

**HPV**, human papillomavirus; numbers in bold indicate *p* values <0.05.

**Table 2:** Univariable and multivariable Cox proportional hazards regression for overall survival and progression-free survival (*n* = 280).

Dependent variable:	Overall survival		Progression-free survival	
Variable:	HR (95% CI)	*p* value	HR (95% CI)	*p* value
Univariable:				
Age at diagnosis (per 5 years)	1.12 (1.02, 1.24)	**0.018**	1.08 (1.00, 1.17)	0.059
Alcohol abuse (vs. No)	1.67 (1.10, 2.52)	**0.015**	1.33 (0.94, 1.90)	0.110
Location		0.749		0.314
Oral cavity vs. oropharynx	1.36 (0.82, 2.26)	0.227	1.30 (0.85, 2.00)	0.232
Larynx vs. oropharynx	1.05 (0.49, 2.23)	0.899	1.80 (1.01, 3.21)	**0.047**
Hypopharynx vs. oropharynx	1.41 (0.48, 4.12)	0.527	0.99 (0.35, 2.83)	0.985
Other vs. oropharynx	0.86 (0.12, 6.42)	0.886	0.61 (0.08, 4.50)	0.630
T stage		**<0.001**		**0.031**
T3 vs. T1-T2	1.52 (0.83, 2.78)	0.179	1.54 (0.96, 2.47)	0.075
T4 vs. T1-T2	2.64 (1.63, 4.30)	**<0.001**	1.70 (1.13, 2.54)	**0.011**
N stage		**<0.001**		**0.036**
N1 vs. N0	1.39 (0.71, 2.72)	0.332	0.95 (0.54, 1.66)	0.851
N2-N3 vs. N0	2.41 (1.53, 3.80)	**<0.001**	1.56 (1.07, 2.28)	**0.020**
Overall stage (AJCC 7^th^ edition)		**<0.001**		0.226
III vs. I-II	1.42 (0.60, 3.38)	0.423	0.97 (0.53, 1.77)	0.917
IV vs. I-II	3.11 (1.55, 6.23)	**0.001**	1.36 (0.85, 2.20)	0.204
Perineural invasion (vs. No)	2.58 (1.61, 4.14)	**<0.001**	1.91 (1.27, 2.88)	**0.002**
Extranodal extension (vs. No)	2.92 (1.73, 4.93)	**<0.001**	2.05 (1.27, 3.30)	**0.003**
Lymphovascular invasion (vs. No)	2.81 (1.73, 4.56)	**<0.001**	1.72 (1.11, 2.66)	**0.016**
Neoadjuvant treatment (vs. No)	1.93 (0.89, 4.19)	0.096	2.84 (1.53, 5.30)	**0.001**
Nonsurgical treatment (vs. No)	0.61 (0.37, 1.02)	0.058	0.87 (0.58, 1.30)	0.502
Adjuvant treatment (vs. No)	1.46 (0.96, 2.22)	0.076	1.07 (0.75, 1.52)	0.714
HPV+ (vs. HPV−)	0.66 (0.41, 1.06)	0.086	0.70 (0.47, 1.04)	0.073
HPV status		0.168		0.099
HPV-16 vs. HPV-Other	2.90 (0.39, 21.5)	0.299	4.83 (0.66, 35.3)	0.121
HPV-negative vs. HPV-other	4.06 (0.56, 29.3)	0.164	6.23 (0.87, 44.7)	0.069
p16+ (vs. p16-/unknown)	0.41 (0.21, 0.82)	**0.011**	0.48 (0.28, 0.82)	**0.008**
Multivariable:				
T stage		—		**0.022**
T3 vs. T1-T2	—	—	1.48 (0.84, 2.61)	0.172
T4 vs. T1-T2	—	—	2.01 (1.22, 3.29)	**0.006**
N stage		—		**0.007**
N1 vs. N0	—	—	1.00 (0.49, 2.01)	0.989
N2-N3 vs. N0	—	—	2.00 (1.27, 3.16)	**0.003**
Overall stage (AJCC 7^th^ edition)		**<0.001**		—
III vs. I-II	1.26 (0.42, 3.76)	0.676	—	—
IV vs. I-II	4.67 (2.01, 10.9)	**<0.001**	—	—
Perineural invasion (vs. No)	2.53 (1.58, 4.07)	**<0.001**	1.60 (1.04, 2.46)	**0.034**
Neoadjuvant treatment (vs. No)	—	—	4.28 (2.07, 8.85)	**<0.001**

**HR**: hazard ratio, **CI**: confidence interval; **HPV**: human papillomavirus; numbers in bold indicate *p* values <0.05.

## Data Availability

The datasets used and/or analyzed during the current study are available from the corresponding author on reasonable request.

## Abbreviations

HPV: Human papillomavirus
qPCR: Quantitative polymerase chain reaction
OS: Overall survival
PFS: Progression-free survival
HNSCC: Head and neck squamous cell carcinomas
TCGA: The cancer genome atlas
OPSCC: Oropharyngeal squamous cell carcinomas
gDNA: Genomic DNA
NTC: No template control
Ct: Threshold cycle
ANOVA”: Analysis of variance
HR: Hazard ratio.

## Supplementary Materials

Supplementary Materials Supplemental Table 1: HPV Primer/probe sets.
