# Supplementary material for: Does HPV Subtype Predict Outcomes in Head and Neck Cancers?
Source: Int J Otolaryngol. 2021 Feb 9;2021:6672373. doi: 10.1155/2021/6672373 (PMC8006754; doi:10.1155/2021/6672373)
Supplement: Supplementary Materials — Supplemental Table 1: HPV Primer/probe sets. [file 6672373.f1.docx]

**Supplemental Table 1.** Summary of HPV primer/probe sets used in study.

| **Primer/ Probe** | **Sequence** | **Product size (nt)** | **Binding position** |
| --- | --- | --- | --- |
| HPV16 Forward | TTGCAGATCATCAAGAACACGTAGA | 110 | 520-544 |
| HPV16 Reverse | GTAGAGATCAGTTGTCTCTGGTTGC |  | 606-630 |
| HPV16 probe | **JOE**-AATCATGCATGGAGATACACCTACATTGCATGA |  | 558-590 |
| HPV18 Forward | CAACCGAGCACGACAGGAACG | 137 | 530-550 |
| HPV18 Reverse | TAGAAGGTCAACCGGAATTTCAT |  | 645-667 |
| HPV18 Probe | **ROX**-AATATTAAGTATGCATGGACCTAAGGCAACATTGCAA |  | 580-616 |
| HPV33 Forward | TAGAGAAACTGCACTGTG | 80 | 540-560 |
| HPV33 Reverse | ATATAAATCTAAAACATATTCCTTT |  | 596-620 |
| HPV33 Probe | **JOE**-AGAGGACACAAGCCAACGTTAA |  | 576-597 |
| HPV35 Forward | AGGTCGGTGTATGTCCTGTTGGAAAC | 123 | 528-551 |
| HPV35 Reverse | CCAAATCTAAAACATAGTCT |  | 627-650 |
| HPV35 Probe | **ROX**-GAGGTGTAAATCATGCATGGAGAAATAA |  | 600-619 |
| HPV56 Forward | AAACATCTAGAGAACCTAGAGA | 68 | 535-556 |
| HPV56 Reverse | ACGTCTTGCAGCGTTGGCACTTTA |  | 580-603 |
| HPV56 Probe | **FAM**-TCTACAGTATAATCATGCATGGTA |  | 558-581 |
| GAPDH Forward | TTCATAACTGTCTGCTTCTCTGCT | 134 | 7583-7606 |
| GAPDH Reverse | TTGAGGCTGTTGTCATACTTCTCA |  | 7694-7717 |
| GAPDH Probe | **CY5**-GGGAGCCAAAAGGGTCATCA |  | 7625-7644 |

**HPV** – human papillomavirus
